# Olive Oil Benefits from Sesame Oil Blending While Extra Virgin Olive Oil Resists Oxidation during Deep Frying

**DOI:** 10.3390/molecules28114290

**Published:** 2023-05-24

**Authors:** Evangelia T. Ioannou, Konstantinos S. Gliatis, Evangelos Zoidis, Constantinos A. Georgiou

**Affiliations:** 1 Chemistry Laboratory, Department of Food Science and Human Nutrition, Agricultural University of Athens, 75 Iera Odos, 118 55 Athens, Greece; evioannou@aua.gr (E.T.I.);; 2 Laboratory of Nutritional Physiology and Feeding, Department of Animal Science, Agricultural University of Athens, 75 Iera Odos, 118 55 Athens, Greece; ezoidis@aua.gr; 3 FoodOmics.GR Research Infrastructure, 118 55 Athens, Greece

**Keywords:** antioxidants, deep frying, olive oil, anisidine value, sesame oil, sesame lignans, total polar compounds

## Abstract

Fresh potatoes were deep-fried in olive oil (OO), extra virgin olive oil (EVOO), and their blends with 5%, 10%, and 20% *v*/*v* sesame oil (SO). This is the first report on the use of sesame oil as a natural source of antioxidants during olive oil deep frying. The oil was evaluated for anisidine value (AV), free fatty acids (FFAs), extinction coefficient (K_232_ and K_270_), Trolox equivalent antioxidant capacity (TEAC), and total phenols (TPs) until the total polar compounds (TPCs) reached 25%. Sesame lignan transformations were monitored through reversed-phase HPLC. While the TPCs in olive oils increased at a steady rate, the addition of 5%, 10%, and 20% *v*/*v* SO delayed TPCs’ formation for 1, 2, and 3 h, respectively. The addition of 5%, 10%, and 20% *v*/*v* SO increased the olive oil frying time by 1.5 h, 3.5 h, and 2.5 h, respectively. The addition of SO to OO reduced the secondary oxidation products’ formation rate. The AV for EVOO was lower than OO and all tested blends, even those with EVOO. EVOO was more resistant to oxidation than OO, as measured by the TPCs and TEAC, while the frying time rose from 21.5 to 25.25 h when EVOO replaced OO. The increase in frying time for OO but not for EVOO, after SO addition, points to a niche market for EVOO in deep frying.

## 1. Introduction

Deep frying is one of the world’s most popular culinary processes, both for industrial and domestic food preparation purposes. During deep frying, food is immersed in hot oil at temperatures of 150 to 190 °C. In the presence of air, many complex reactions take place such as oxidation, hydrolysis, and polymerization [1,2,3]. These reactions influence the quality of the final product, such as the flavor, texture, shelf life of the oil, and nutrient composition, with potential adverse effects on human health [4]. The type of frying oil, its chemical composition, and its physical and physicochemical properties are major parameters that influence the chemical reactions and determine the performance of the frying oil against oxidation and decomposition [5].

Over the ages, olive oil has been widely produced and consumed in Mediterranean countries, with it being the main lipid source in the Mediterranean diet. Its beneficial properties are associated with fatty acid composition, phenolic antioxidants, and other minor compounds that make olive oil a very interesting option among oils and fats [6,7]. Extra virgin olive oil exhibits high resistance to oxidation in comparison with other vegetable oils, and it is well known for its very good sensory and health properties [8,9]. Olive oil is resistant to degradation under domestic frying conditions, independently of its category label [10]. Olive oil’s naturally occurring antioxidants play a significant role in the thermal stability during deep frying [11]. Synthetic and natural antioxidants can be added to prevent or minimize the oxidative deterioration of the oil. The most commonly used antioxidants are butylated hydroxy anisole (BHA), butylated hydroxytoluene (BHT), propyl gallate (PG), and tert-butylhydroquinone (TBHQ). However, the use of synthetic antioxidant additives is regulated in most countries because of concerns regarding their long-term health effects. Natural components in foods with radical scavenging or antioxidant activity have attracted interest as alternatives to synthetic antioxidants. The addition of sources of natural antioxidants can possibly be used to improve olive oil’s resistance to the formation of primary and secondary oxidation products without making considerable changes to their natural composition. Blending different types of vegetable oils may help to extend the thermal stability and nutritional profile of frying oils [12]. Sesame oil demonstrates higher oxidative stability than other vegetable oils [2,13]. The study of this superior oxidative stability has mainly been focused on sesame lignans, which are present in small amounts in sesame oil. Sesamin and sesamolin are the major lignans found in sesame seeds [14,15]. When sesame seeds are roasted at a high temperature, sesamolin degrades into sesamol [16]. Sesamol is reported to possess higher radical scavenging activity compared to sesamin and sesamolin [17]. The significant stability of sesame oil could be related with the continuous generation of sesamol from the degradation of sesamolin during thermal oxidation, rather than the initial antioxidant content [18]. 

In one study, the addition of sesame lignans in oils during frying increased sesamol and decreased sesamolin, while sesamin was rather resistant to heat. Sesame lignans could have applications as natural antioxidants in the edible oil and food industry [19]. Other studies suggest that lignan compounds in sesame oil are effective antioxidants in deep fat frying due to their high stability and efficacy [2,20]. The addition of roasted sesame oil, as a natural source of antioxidants, prolonged the oil’s heat stability and shelf life. Moreover, when the roasted sesame oil concentration increased, the antioxidant capacity of frying oils increased. The proper blending of high polyunsaturated sunflower oil with sesame oil can produce oil blends of high nutritional value and enhanced stability for home cooking and deep frying [21]. 

Consumers’ perception of the health benefits of olive oil creates a niche market for olive oil for deep frying, despite its high price. Consumers nowadays may have a different perception of extra virgin olive oil, recognizing its superiority over olive oil. This creates the need to further study these two olive oil products during deep frying and assess the benefits of adding antioxidants as blends with natural products that will not deteriorate consumers’ perception of the health benefits of olive oil. Although olive oil’s deep-frying characteristics have been studied, we were not able to find any study with sesame oil admixtures. In this study, we describe the effect of adding virgin sesame oil as a source of natural antioxidants at different levels to extra virgin olive oil and olive oil during domestic deep frying. Sesame lignan changes in the oil samples during deep frying and the correlation between the sesame oil antioxidant activity and its efficacy in retarding the oxidative deterioration of olive oil and extra virgin olive oil were investigated. In order to evaluate the oxidation progress, we monitored the total polar compounds, anisidine value, free fatty acids, extinction coefficient, total phenols, and antioxidant activity.

## 2. Results and Discussion 

### 2.1. Total Polar Compounds

**Figure 1:**
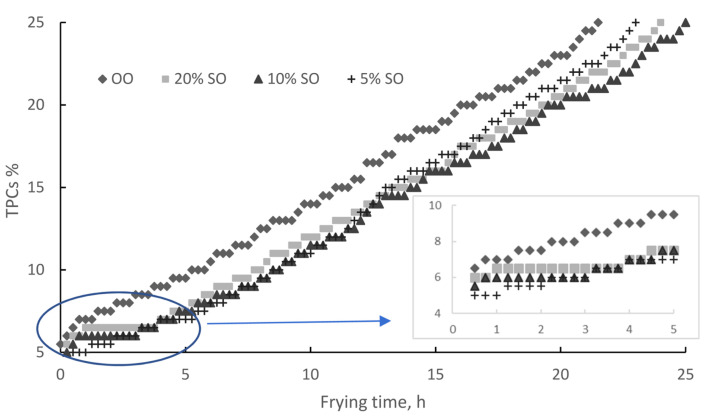
Total polar compounds during deep frying: olive oil and sesame oil blends.

**Figure 2:**
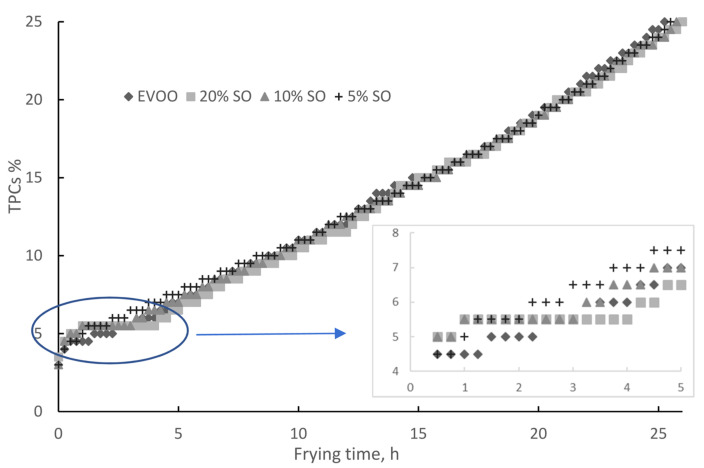
Total polar compounds during deep frying: extra virgin olive oil and blends with sesame oil.

The formation of polar compounds indicates oil deterioration and is strongly related to primary and secondary oxidation during frying. Total polar compounds are considered one of the most objective indicators in evaluating the deterioration of deep-frying oils [22,23]. In several countries, the rejection value for TPCs has been set at 25% by weight for frying oils [24]. TPCs increased linearly, as shown in Figure 1 for OO and Figure 2 for EVOO. The OO frying time increased from 21.5 to 23, 25, and 24 h upon the addition of 5, 10, and 20% SO, respectively. During this procedure, the formation of TPCs was slightly delayed when sesame oil was added. The delay was proportional to the SO concentration, lasting 1, 2, and 3 h for 5, 10, and 20% SO, respectively. Then, TPCs increased at a steady rate of 1% per hour for all three blends, which is lower in comparison to 1.5% for pure OO.

The EVOO frying time was not increased by SO blending and was approximately the same (25–26 h) for all blends. This is due to the higher antioxidant content of EVOO, indicating the higher nutritional value and health benefits of EVOO in comparison to OO. The TPC formation rate for EVOO did not decrease upon the addition of SO; it was always 1% per hour. A slight delay in the formation of TPCs upon 10 and 20% SO addition to EVOO was also detected, while 5% had no effect. After this delay, TPCs increased at a steady rate of 1% per hour (Figure 2).

### 2.2. Lignans

**Figure 3:**
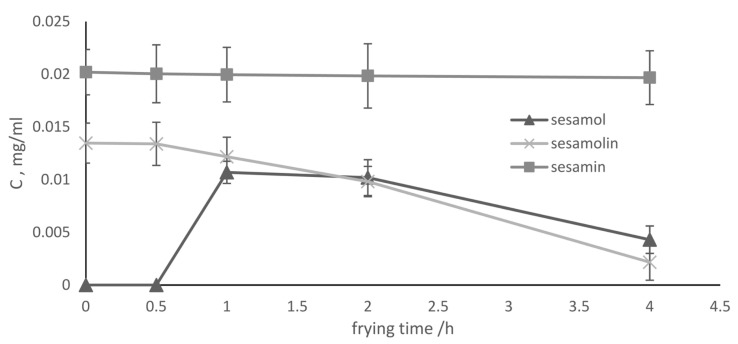
Sesame lignans during deep frying using 20% *v*/*v* sesame oil in olive oil.

Large amounts of sesamol are produced from sesamolin during frying, contributing to oil stability [16,25]. Chatzos and Georgiou [26] reported that radical scavenging activity increases during virgin sesame oil heating, in contrast to all other seed oils in their study. This paradox is attributed to the fact that sesamol has higher antioxidant activity than its precursor, sesamolin [17]. We followed the decomposition of sesamolin to sesamol during the domestic deep frying of potatoes in admixtures with olive oil through reverse-phase HPLC. Initially, only sesamin and sesamolin were detected (Figure 3). After 1h of frying, sesamol was detected and sesamolin decreased by 18%. After 2 h of deep frying, both sesamol and sesamolin decreased by 5% and 38%, respectively. After 4 h, sesamol and sesamolin decreased further by 60% and 70%, respectively. A similar study on sesame oil by Hemalatha and Ghafoorunissa is in accordance with our study, reporting the maximum concentration of sesamol after one hour followed by a gradual decrease for one more hour [27].

As mentioned in Section 3.1, the TPCs after one hour of frying remained constant at the same time as when the sesamol concentration peaked. Then, after four hours, when sesamol was depleted, the TPCs increased again, while all blends exhibited similar behavior. This points to a beneficial role of sesame oil addition. The delay of TPC formation correlates to sesamolin concentrations: higher concentrations increase the delay time. This finding is in agreement with previous research, where the antioxidant capacity of sesamol may have been influenced by the concentration [28].

### 2.3. Changes in Anisidine Value

Deep frying promotes secondary oxidation products, mostly conjugated dienals and 2-alkenals, which are more stable during the heating process. Thus, the AV is an essential and reliable test to measure oil oxidation [29]. These compounds are accessed through the anisidine value. The AV increased significantly for both the OO and the blends with SO during the initial 12h. Then, the AV increased at a lower rate. It should be noted that the blends always scored lower than OO (Table 1).

The AV significantly increased for EVOO and all its SO blends during the initial 12 h (Table 1). At the same time, the blends scored higher in the AV. Then, the AV increased at a lower rate, while the blends still scored higher. It is interesting to note that secondary oxidation products were lower in EVOO throughout the study. This points out that the antioxidant compounds therein are much more efficient than sesame oil.

### 2.4. Free Fatty Acids

Hydrolysis in fats and oils results in the formation of free fatty acids, mono- and di-glycerides, and glycerol, inducing oxidative degradation and contributing to shelf life reduction [2]. The free fatty acid content increases during frying [30]. 

The FFA content in the OO sample was 0.71%, which is within the legally accepted limit of 1%. After 12h of frying, the FFA content in OO and the three blends increased to 1.44, 1.52, 1.47, and 1.59, respectively, as shown in Table 2. The total frying time was 21.5, 23, 25, and 24 h for OO and the three blends. The end FFA values were 1.9, 2.3, 2.8, and 3.0%, respectively. Although the frying time increased due to SO addition, it was accompanied by a higher FFA content, which could have a negative organoleptic impact or even surpass the maximum value set in specific countries (2.5%) [24]. 

The FFA content in EVOO was 0.67%, which is within the legally accepted limit of 0.8% established by the Commission Delegated Regulation (EU 2015/1830). After 12 h of deep frying, the FFA content in EVOO and the three blends increased to 1.29, 1.30, 1.45, and 1.41%, respectively, as shown in Table 2. The total frying time was 25, 25.25, 25.5, and 26 h for EVOO and the three blends. The end FFA values were 2.2, 1.8, 2.4, and 2.4%, respectively. The frying time was accompanied by a higher FFA content without exceeding the maximum value set in specific countries (2.5%) [24].

### 2.5. Extinction Coefficients: K_232_ and K_270_

When polyunsaturated fatty acids are oxidized, ultraviolet absorption increases. The changes in ultraviolet absorption at 232 nm are associated with the formation of conjugated dienes of polyunsaturated fatty acids, while changes at 270 nm are associated with the formation of conjugated trienes and carbonyl compounds. OO’s and EVOO’s values of K_232_ and K_270_ are shown in Table 2 and Table 3, respectively. The values for OO, EVOO, and their blends with SO increased during frying. There were no evident differences between the types of oil or between the blends. The increase was not related to the amount of SO. Moreover, no difference between OO and EVOO was recorded.

### 2.6. Changes in Antioxidant Capacity

Lipid oxidation is a free radical reaction that is strongly modulated by synthetic and natural antioxidant compounds. During deep frying, antioxidant compounds are consumed, resulting in a lower score in antioxidant capacity tests. Measured by the radical scavenging ability while reacting with a relatively stable radical such as 2,2-diphenyl-1-picrylhydrazyl (DPPH) [31]. 

In our study, the total antioxidant capacity was expressed as the Trolox equivalent antioxidant capacity (TEAC), defined as the mmol Trolox/kg of oil. The OO blends with SO initially had higher TEAC values than OO, which were proportional to the percentage of SO in the blend (Table 2). After 12 h of deep frying, all oils reached extremely low TEAC values. This comes in accordance with Kalantzakis et al.’s study, where olive oil samples practically lost their radical scavenging activity after 5 h of heating at 180 °C [32]. Although the oil samples were not expected to present any radical scavenging activity at the end of the frying experiment, the TEAC values were elevated for all samples (Figure S1a). This is an artifact explained by the reaction of DPPH with aldehydic compounds that are the end products of lipid oxidation and are produced in high concentrations during the late stage of deep frying [33].

The antioxidant capacity of EVOO was not increased that much with the addition of SO, as with OO (Table 2 and Table 3, zero frying time). The antioxidant capacity of EVOO after 12 h of deep frying decreased by 84%. This improved to 72% upon 20% SO addition (Table 3). In a similar way as was said for OO above, the TEAC values increased after 25 h of deep frying (Figure S1b).

### 2.7. Changes in Total Phenols

Phenolics are the major health-promoting compounds in olive oil and the Mediterranean diet [34]. It is, therefore, important to follow the evolution of phenolics during deep frying to assess the potential health benefits of using olive oil. 

The initial phenolic content of OO was 55.96 mg/Kg. The total phenols suffered a significant decrease for OO and all its blends with SO after 12 h of deep frying, going almost to zero (Figure S2).

The initial amount of phenolics in EVOO was 127.0 mg/Kg. After 12 h of deep frying, EVOO and all its blends with SO retained 30% of their phenolic content. After 25 h, there were no phenolic compounds left (Figure S3).

### 2.8. Fatty Acid Composition

The fatty acid compositions reflected the high proportion of oleic acid in OO (74.6%) and EVOO (75%). In contrast to olive oils, the major fatty acid in SO is linoleic (41.5%), followed by oleic (31.9%), palmitic (14.8%), and stearic (9%) acids. The fatty acid compositions of SO, OO, and EVOO were in agreement with values from the literature [35,36].

## 3. Materials and Methods

### 3.1. Reagents

Isooctane, isopropanol, glacial acetic acid, chloroform, ethyl acetate, acetone, diethyl ether, ethanol, n-hexane and methanol of analytical grade, sodium acetate of pro-analysis grade, and potassium hydroxide pellets for analysis were obtained from Merck, Darmstadt, Germany.

p-anisidine 99%, 2,2-Diphenyl-1-picrylhy-drazyl (DPPH) 90%, and 6-hydroxy-2,5,7,8-tetramethylchroman-2-car-boxylic acid (Trolox) 97% were purchased from Sigma-Aldrich (St. Louis, MO, USA). Gallic acid monohydrate was supplied from Riedel-de Haen (Seelze, Germany). LC-MS-grade methanol and CDCl3 98% D were purchased from Fluka. Sesamol was purchased from Sigma Chemical Co. (St. Louis, MO, USA).

### 3.2. Oil Samples

A commercial extra virgin olive oil (EVOO); a commercial blend of refined olive oil and virgin olive oil labeled as olive oil (OO) purchased from Minerva S.A. (Athens, Greece); and commercial virgin sesame oil (SO) purchased from Haitoglou Bros S.A. (Thessaloniki, Greece) were used for the frying experiments. The oils were purchased from local stores in Athens, Greece in sealed and marked commercial containers.

### 3.3. Frying Process

A 2.5 L domestic deep-frying electric fryer (KENWOOD DF520) was used, where the temperature was regulated at 170 ± 5 °C. Fresh potato strips (7 cm × 0.5 cm × 0.5 cm), of Spunta variety cultivated in Greece, were deep-fried in 70 g batches. The batches were fried at 9 min intervals for 12 h per day for two consecutive days, without oil replenishment, until the oil was discarded. The end-of-frying assay and oil rejection point were determined by the value of total polar compounds (max. 25%), according to the regulation of frying fats and oils in most European Countries [24]. The frying experiment was planned in such a way as to simulate a continuous/prolonged deep-frying process.

### 3.4. Oil Sampling

After each frying session, lasting 15 min, the TPCs were assessed directly on the hot oil. Extinction coefficients (K_232_ and K_270_), antioxidant capacity (AC), free fatty acids (FFAs), and total phenols (TPs) were assessed by removing a 6 mL sample: (a) right after the thermal equilibration of the oil to 170 ± 5 °C before frying the potatoes, (b) at 12 h, and (c) at the time when the TPCs reached 25% and the oil was rejected. The anisidine value (AV) was assessed by removing 0.5 mL sample at regular intervals. The samples were placed in screw-cap glass vials and were immediately stored in the freezer until analysis. To monitor sesame lignan transformations, 2 mL oil samples were removed before starting and after 1 h, 2 h, and 4 h.

### 3.5. Total Polar Compounds

TPC estimation was based on the dielectric constant changes, measured directly on the hot oil with a Testo 270 sensor (Testo SE & Co. KGaA, Titisee-Neustadt, Germany). Before measuring, about 5 min was allowed after removing the fried potatoes until there were no more bubbles rising. The sensor took about 1 min to obtain a stable reading. TPC % content along with the temperature were displayed on the screen of the sensor. Sensor calibration was performed through the oil, supplied by the Testo 270 manufacturer, right before the analysis. The sensor was cleaned with warm water and neutral detergent and dried well between measurements.

### 3.6. HPLC Analysis

Sesamin and sesamolin were isolated and crystallized from sesame oil, as described by Hemalatha and Ghafoorunissa [26]. Sesamin and sesamolin were characterized with mass spectrometry and ^1^H-NMR and ^13^C-NMR spectrometry. Sesamol was purchased from Sigma Chemical Co. (St. Louis, MO, USA). HPLC analysis was carried out with an Agilent Technologies 1100 series diode array detector. Sesame lignans were analyzed as described by Wen-Huey Wu [37], using HPLC equipped with a Supelco Analytical HPLC column (Discovery HS C18) with a length of 250 mm, internal diameter of 4.6 mm, and particle size of 5 μm. The mobile phase was a mixture of methanol–deionized water (70:30, *v*/*v*) at a flow rate of 0.8 mL/min and the column temperature was maintained at 25 °C. The absorption at 290 nm was monitored. Twenty-microliter aliquots of oils, dissolved in chloroform (0.5 mg/mL), were injected for analysis. The retention times for standard sesamol, sesamin, and sesamolin were 4.6, 15.6, and 20.8 min, respectively (Figures S4–S9, Table S2).

### 3.7. Analytical Methods

The AV was determined according to the modified European Pharmacopoeia 5.0 method. The oil samples were dissolved in isooctane and then allowed to react for ten minutes with a 2.5 g/L p-anisidine solution in acetic acid. The absorbance value was measured at 350 nm using the Cary 60 Scan UV–visible spectrophotometer. The spectrophotometer gives the average absorbance of three readings.

The extinction coefficients (K_232_ and K_270_) and free acidity of the oils were determined according to the analytical methods described in European Commission Regulations (Commission Regulation (EEC) No.2568/91) [38]. The total antioxidant capacity was determined through DPPH radical. A 65 mg oil sample was added to 4 mL of a 1.3 × 10^−4^ M solution of DPPH in ethyl acetate. Then, the mixture was shaken vigorously and left in darkness for 1 h. Finally, the absorbance of the mixture was measured against ethyl acetate (blank) at 515 nm with a spectrophotometer (Cary 60 Scan UV–visible spectrophotometer). The total antioxidant capacity was expressed as Trolox equivalent antioxidant capacity (TEAC), defined as the mmol Trolox/kg of oil [26]. The total phenols in the methanolic extract of the oils were determined colorimetrically at 765 nm with Folin–Ciocalteu reagent according to Capannesi et al. [39]. Gallic acid standard solutions were used for calibration (r = 0.9998).

### 3.8. Fatty Acid Analysis

The sample (0.1 g) was dissolved in 2 mL of heptane and shaken. The sample was then transesterified with 0.2 mL of 2 N methanolic potassium hydroxide solution and vigorously shaken (Commission Regulation (EEC) No.2568/91) [37]. Methyl esters in the upper layer were assessed with GC–FID. GC was performed on a 30 m × 0.25 mm i.d. × 0.25 μm film HP-5MS capillary column using a Hewlett Packard (Waldbronn, Germany) 5890 gas chromatograph connected to a flame ionization detector (FID). The GC conditions used were as follows: injection volume, 1 μL; split injection, 50:1 at 220 °C; and oven temperature ramped to 270 °C at 5 °C min^−1^ held for 5 min, giving a total run time of 55 min. The helium gas carrier was held at a constant flow rate of 1 mL min^−1^, whilst the detector was set at a temperature of 290 °C.

## 4. Conclusions

Blending olive oil with sesame oil decreases secondary oxidation products throughout the frying period until its rejection at 25% total polar compounds. Through blending, the useful frying period increases from 21.5 h to 25 h in proportion to the amount of sesame oil, reaching the frying time of extra virgin olive oil, which does not benefit from blending.

Blending extra virgin olive oil, in contrast to olive oil, increases secondary oxidation products. It is clear that the natural antioxidant compounds in extra virgin olive oil are more efficient at preventing deep-frying oxidative damage than sesame oil blending. 

In summary, sesame oil addition increases olive oil’s frying time but is not beneficial for extra virgin olive oil. This points to a niche market for extra virgin olive oil in deep frying. 

We report, for the very first time, that the formation of total polar compounds during olive oil deep frying is decreased through sesame oil blending, pointing to a need for further research. 

Olive oil is very much appreciated in the Mediterranean and Greek diets, with the biggest benefit coming from phenolic antioxidants. Sesame oil addition did not have any effect on protecting olive oil phenolics during frying. Our study showed that olive oil lost all phenolics after 12 h, while extra virgin olive oil retained around 30%. This result could evolve after further research to a guideline on the use of extra virgin olive oil for deep frying so that the beneficial phenolics are not depleted. Such a guideline could help branding extra virgin olive oil as a health-promoting frying oil. We envisage that deep frying with extra virgin olive oil should not continue until the total polar compounds reach 25%, but should stop much earlier to spare phenolic antioxidants. 

## Figures and Tables

**Table 1:** Changes in AV during deep frying. Olive oil, extra virgin olive oil, and blends with sesame oil.

Time	Frying Cycle	Anisidine Value							
		OO	OO Blends with SO			EVOO	EVOO Blends with SO		
			5%	10%	20%		5%	10%	20%
0	1	26.3	18.9	12.2	23.5	16.0	20.4	20.1	36.6
0.5	3	30.7	21.9	14.3	24.0	19.7	30.9	28.7	40.7
1	5	45.8	26.7	15.3	24.3	21.2	36.6	34.9	42.0
1.5	7	52.1	29.6	19.4	33.4	23.6	44.7	38.5	41.7
2.5	11	54.0	38.1	26.9	36.1	29.1		45.7	45.6
4	17	55.3	48.8	37.9	46.0	41.4	54.2	45.9	56.1
4.5	19	52.5	48.9	41.5	48.7	47.2	54.6	46.4	59.65
9.5	39	95.9		63.0	76.7	71.8		66.9	74.9
12	49	97.8	85.7	69.5	80.6	64.3	67.6	68.5	90.9
14	57	102.0	90.0	75.9	90.3	76.3	75.2	88.4	97.1
16	65	109.2	94.3	79.4	96.4	77.8	86.7	90.3	107.2
18	73	113.4		84.5	109.3	92.2	96.9	97.9	102.3
21.25	86	128.2							
23	93		114.1	96.2	124.6	97.1	105.3	100.0	122.5
24	97		124.9	102.0	126.8	111.9	115.9	117.8	
24.75	100					114.7	119.6		
25	101			112.3				119.9	129.2
26	105							121.6	129.9

OO: olive oil, EVOO: extra virgin olive oil, SO: sesame oil.

**Table 2:** Olive oil and blends with sesame oil, changes in FFA, extinction coefficients, and TEAC value.

Frying Time (h)	FFA	K_232_	K_270_	TEAC (mmol/Kg)
OO				
0	0.71 ± 0.03	1.55	0.20	0.91 ± 0.01
12	1.44 ± 0.08	3.91	1.77	0.15 ± 0.02
21.5	1.91 ± 0.05	3.99	1.86	0.26 ± 0.02
5% SO				
0	0.69 ± 0.00	1.19	0.21	1.10 ± 0.01
12	1.52 ± 0.04	3.91	1.08	0.02 ± 0.02
23	2.34 ± 0.05	3.90	1.21	0.60 ± 0.02
10% SO				
0	0.58 ± 0.08	1.43	0.26	1.29 ± 0.01
12	1.47 ± 0.05	2.24	1.29	0.099 ± 0.01
25	2.81 ± 0.1	4.15	1.74	0.53 ± 0.03
20% SO				
0	0.41 ± 0.01	2.09	0.36	1.37 ± 0.01
12	1.59 ± 0.06	2.16	1.61	0.18 ± 0.02
24	3.03 ± 0.04	4.13	2.27	0.92 ± 0.03

OO: olive oil, SO: sesame oil.

**Table 3:** Extra virgin olive oil and blends with sesame oil, changes in FFA, extinction coefficients, and TEAC value.

Frying Time (h)	FFA	K_232_	K_270_	TEAC (mmol/Kg)
EVOO				
0	0.67 ± 0.01	1.22	0.13	1.91 ± 0.02
12	1.29 ± 0.05	3.63	0.89	0.32 ± 0.01
25	2.21 ± 0.07	2.94	0.79	0.49 ± 0.03
5% SO				
0 h	0.67 ± 0.00	2.13	0.25	1.63 ± 0.01
12 h	1.30 ± 0.08	3.39	1.21	0.34 ± 0.02
25.25 h	1.79 ± 0.05	3.47	1.27	0.44 ± 0.02
10% SO				
0h	0.63 ± 0.03	2.06	0.19	2.1 ± 0.02
12h	1.45 ± 0.04	3.11	1.31	0.46 ± 0.02
25.5 h	2.44 ± 0.07	2.35	1.41	0.68 ± 0.03
20% SO				
0 h	0.63 ± 0.01	2.03	0.36	2.03 ± 0.01
12 h	1.41 ± 0.12	2.77	1.55	0.56 ± 0.02
26 h	2.42 ± 0.08	4.06	1.92	0.94 ± 0.02

EVOO: extra virgin olive oil, SO: sesame oil.

## Data Availability

Data will be made available on request.

## Supplementary Materials

The following supporting information can be downloaded at: https://www.mdpi.com/article/10.3390/molecules28114290/s1, Figure S1: TEAC (mmol/Kg) values during deep frying (a) OO, (b) EVOO and the blends with SO; Figure S2: Changes in total phenols (mg GA/Kg oil) during deep frying OO and the blends with SO; Figure S3: Changes in total phenols (mg GA/Kg oil) during deep frying EVOO and the blends with SO; Figure S4: HPLC chromatogram of a mixed standard solution of sesamol, sesamin, and sesamolin; Figure S5: HPLC chromatogram of blend of 20% *v*/*v* sesame oil and olive oil before frying; Figure S6: HPLC chromatogram of blend of 20% *v*/*v* sesame oil and olive oil after 30 min frying; Figure S7: HPLC chromatogram of blend of 20% *v*/*v* sesame oil and olive oil after 1 h of frying; Figure S8: HPLC chromatogram of blend of 20% *v*/*v* sesame oil and olive oil after 2 h of frying; Figure S9: HPLC chromatogram of blend of 20% *v*/*v* sesame oil and olive oil after 4 h of frying; Figure S10: mass spectrum of sesamolin; Figure S11: 1H-NMR spectrum of sesamolin; Figure S12: 1H-NMR spectrum of sesamin; Table S1: total polar compounds during deep frying with olive oil (OO), extra virgin olive oil (EVOO), and blends with sesame oil (SO); Table S2: The retention time, standard curve, LOD, and LOQ for sesame lignans.
