# Supplementary material for: Olive Oil Benefits from Sesame Oil Blending While Extra Virgin Olive Oil Resists Oxidation during Deep Frying
Source: Molecules. 2023 May 24;28(11):4290. doi: 10.3390/molecules28114290 (PMC10254163; doi:10.3390/molecules28114290)
Supplement: Supplementary file 1 [file molecules-28-04290-s001.zip › molecules-2396057-supplementary.pdf]

Table S1: Total Polar Compounds during deep frying Olive oil (OO), Extra Virgin Olive Oil (EVOO) and blends with sesame oil (SO).

Time/h	Frying Cycle	TPCs %							
		OO	OO blends with SO			EVOO	EVOO blends with SO		
			5%	10%	20%		5%	10%	20%
0	1	5.5	3.5	3.5	4	3	3	3	3.5
0.25	2	6	4.5	5	5.5	4	4.5	4.5	4.5
0.5	3	6.5	5	5.5	6	4.5	4.5	5	5
0.75	4	7	5	6	6	4.5	5	5	5
1	5	7	5	6	6.5	4.5	5.5	5.5	5.5
1.25	6	6.5	5.5	6	6.5	4.5	5.5	5.5	5.5
1.5	7	7	5.5	6	6.5	5	5.5	5.5	5.5
1.75	8	7.5	5.5	6	6.5	5	5.5	5.5	5.5
2	9	7.5	5.5	6	6.5	5	6	5.5	5.5
2.25	10	8	6	6	6.5	5	6	5.5	5.5
2.5	11	8	6	6	6.5	5.5	6	5.5	5.5
2.75	12	8	6	6	6.5	5.5	6	5.5	5.5
3	13	8.5	6	6	6.5	5.5	6.5	5.5	5.5
3.25	14	8.5	6.5	6.5	6.5	5.5	6.5	6	5.5
3.5	15	8.5	6.5	6.5	6.5	6	6.5	6	5.5
3.75	16	9	6.5	6.5	6.5	6	7	6.5	5.5
4	17	9	7	7	7	6	7	6.5	6
4.25	18	9	7	7	7	6.5	7	6.5	6
4.5	19	9.5	7	7	7.5	6.5	7.5	7	6
4.75	20	9.5	7	7.5	7.5	7	7.5	7	6.5
5	21	9.5	7	7.5	7.5	7	7.5	7	6.5
5.25	22	10	7	7.5	8	7	8	7.5	7
5.5	23	10	7.5	8	8	7.5	8	7.5	7
5.75	24	10	7.5	8	8.5	7.5	8	7.5	7
6	25	10.5	8	8	8.5	7.5	8.5	8	7.5
6.25	26	11	8	8.5	9	8	8.5	8	7.5
6.5	27	11	8	8.5	9	8	8.5	8.5	8
6.75	28	11	8.5	8.5	9	8.5	9	8.5	8
7	29	11.5	8.5	8.5	9.5	8.5	9	8.5	8.5
7.25	30	11.5	9	9	9.5	9	9	9	8.5
7.5	31	11.5	9	9	9.5	9	9.5	9	8.5
7.75	32	12	9	9	10	9	9.5	9	9
8	33	12.5	9.5	9.5	10	9.5	9.5	9.5	9
8.25	34	12.5	9.5	9.5	10.5	9.5	10	9.5	9
8.5	35	13	10	10	11	9.5	10	9.5	9.5
8.75	36	13	10	10	11	10	10	10	9.5
9	37	13	10.5	10.5	11	10	10	10	9.5
9.25	38	13	10.5	10.5	11.5	10	10.5	10	10
9.5	39	13.5	11	11	11.5	10.5	10.5	10.5	10
9.75	40	14	11	11	12	10.5	10.5	10.5	10
10	41	14	11	11.5	12	11	11	11	10.5
10.25	42	14	11.5	11.5	12	11	11	11	10.5

10.5	43	14.5	11.5	11.5	12.5	11	11	11	11
10.75	44	14.5	12	12	12.5	11.5	11.5	11.5	11
11	45	15	12	12	13	11.5	11.5	11.5	11
11.25	46	15	12	12	13	11.5	12	12	11.5
11.5	47	15	12.5	12.5	13	12	12	12	11.5
11.75	48	15.5	13	12.5	13.5	12	12.5	12	11.5
12	49	15.5	13.5	13	13.5	12	12.5	12.5	11.5
12.25	50	16.5	13.5	13.5	14	12.5	12.5	12.5	12
12.5	51	16.5	14	14	14	13	13	13	12.5
12.75	52	16.5	14.5	14	14.5	13	13	13	12.5
13	53	17	15	14.5	14.5	13.5	13	13	13
13.25	54	17	15	14.5	15	14	13.5	13.5	13
13.5	55	18	15.5	14.5	15	14	13.5	13.5	13.5
13.75	56	18	15.5	14.5	15	14	13.5	13.5	13.5
14	57	18	16	15	15.5	14.5	14	14	14
14.25	58	18.5	16	15	15.5	14.5	14	14	14.5
14.5	59	18.5	16	15.5	15.5	14.5	14.5	14.5	14.5
14.75	60	18.5	16.5	16	16	15	14.5	14.5	14.5
15	61	18.5	16.5	16	16	15	14.5	14.5	15
15.25	62	19	17	16	16	15	15	15	15
15.5	63	19	17	16	16.5	15	15	15	15
15.75	64	19.5	17	16.5	17	15.5	15.5	15	15.5
16	65	20	17.5	16.5	17.5	15.5	15.5	15.5	15.5
16.25	66	20	17.5	16.5	17.5	15.5	15.5	15.5	16
16.5	67	20	18	17	17.5	16	16	16	16
16.75	68	20.5	18	17	18	16	16	16	16
17	69	20.5	18.5	17	18	16	16.5	16.5	16
17.25	70	20.5	19	17.5	18	16.5	16.5	16.5	16.5
17.5	71	21	19	17.5	18.5	16.5	16.5	16.5	16.5
17.75	72	21	19.5	18	18.5	17	17	17	16.5
18	73	21	19.5	18	19	17	17	17	17
18.25	74	21.5	20	18.5	19	17	17.5	17.5	17
18.5	75	21.5	20	18.5	19	17.5	17.5	17.5	17.5
18.75	76	22	20.5	19	19.5	18	17.5	17.5	17.5
19	77	22	20.5	19	19.5	18	18	18	18
19.25	78	22.5	21	19.5	20	18.5	18	18.5	18
19.5	79	22.5	21	20	20	18.5	18.5	18.5	18.5
19.75	80	23	21	20	20.5	19	18.5	18.5	18.5
20	81	23	21.5	20	20.5	19	19	19	19
20.25	82	23	21.5	20.5	21	19.5	19.5	19	19
20.5	83	23.5	22	20.5	21	19.5	19.5	19.5	19.5
20.75	84	24	22	20.5	21.5	20	19.5	19.5	20
21	85	24.5	22.5	20.5	21.5	20	20	20	20
21.25	86	24.5	22.5	21	22	20.5	20	20	20
21.5	87	25	22.5	21	22	20.5	20.5	20.5	20.5
21.75	88		23	21	22	21	20.5	20.5	20.5
22	89		23.5	21.5	22.5	21.5	21	21	20.5
22.25	90		23.5	21.5	22.5	21.5	21	21	21

22.5	91		24	22	23	22	21.5	21.5	21
22.75	92		24.5	22	23.5	22	21.5	21.5	21.5
23	93		25	22.5	23.5	22.5	22	22	21.5
23.25	94			23	24	22.5	22.5	22.5	22
23.5	95			23.5	24	23	22.5	22.5	22
23.75	96			23.5	24.5	23	23	23	22.5
24	97			24	25	23.5	23	23	23
24.25	98			24		23.5	23.5	23.5	23
24.5	99			24		24	23.5	23.5	23.5
24.75	100			24.5		24.5	24	23.5	23.5
25	101			25		24.5	24	24	24
25.25	102					25	24.5	24	24
25.5	103						25	24.5	24.5
25.75	104							25	24.5
26	105								25

Table S2. The retention time, standard curve, LOD and LOQ for sesame lignans.

Sesame lignans	Retention time(min)	Regression line		LOD	LOQ
		Standard curve equation	Correlation (R^2)	(mg/mL)	(mg/mL)
sesamol	4,58	y= 0.00241x + 0.00502	0.994	0.002	0.009
sesamin	15,55	y=0.00281x +0.00036	0.9997	0.005	0.02
sesamolin	20,77	y=0.00237x +0.0098	0.99	0.005	0.01

(a)

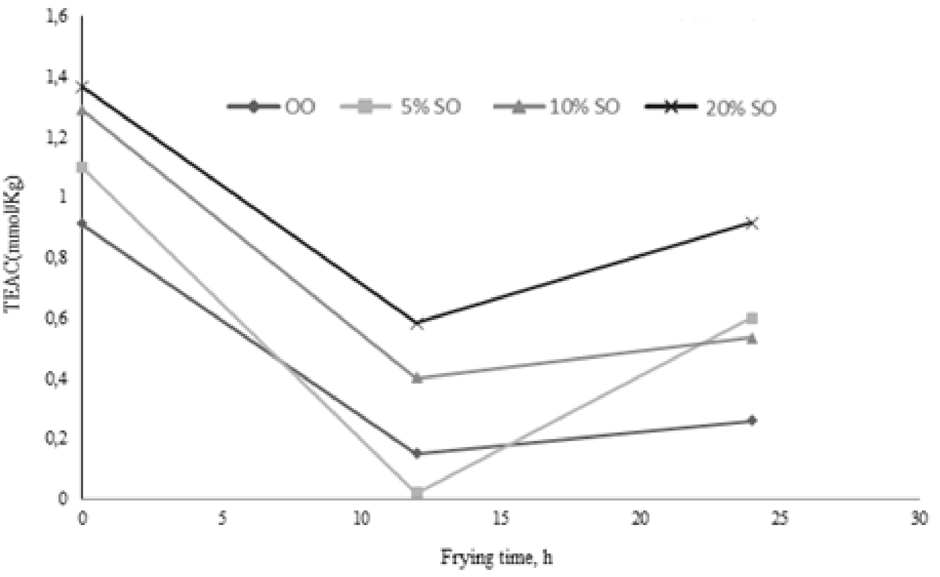

(b)

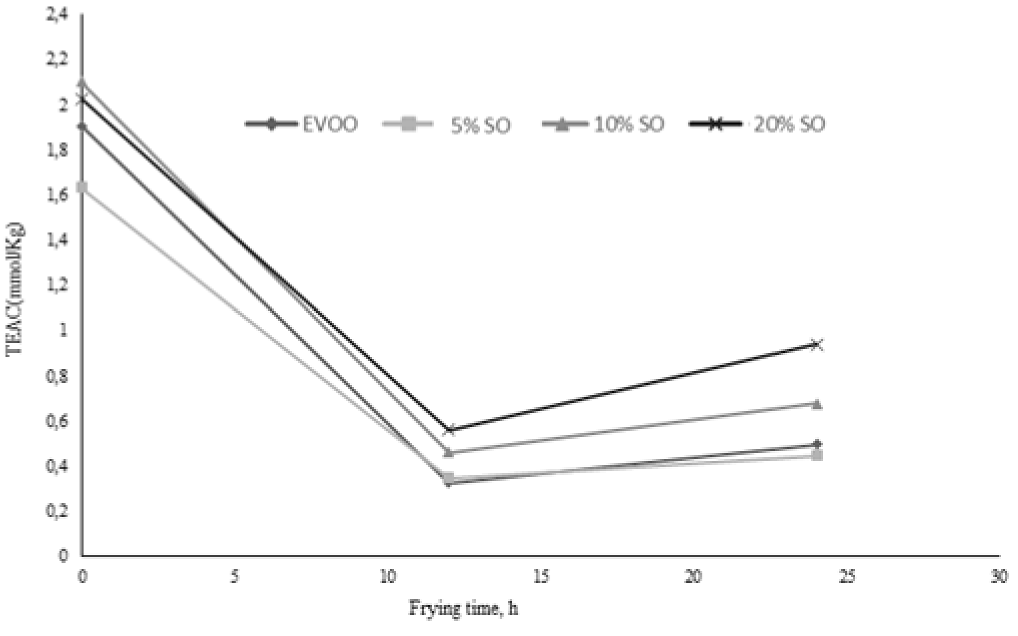

Figure S1: TEAC values (mmol/Kg) during deep frying: (a) OO, (b) EVOO blends with SO

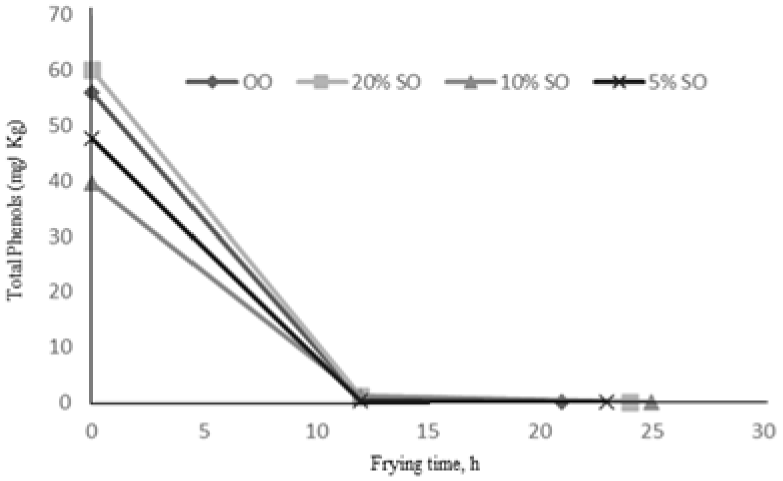

Figure S2: Total phenols (mg GA/Kg oil) during deep frying: OO and blends with SO

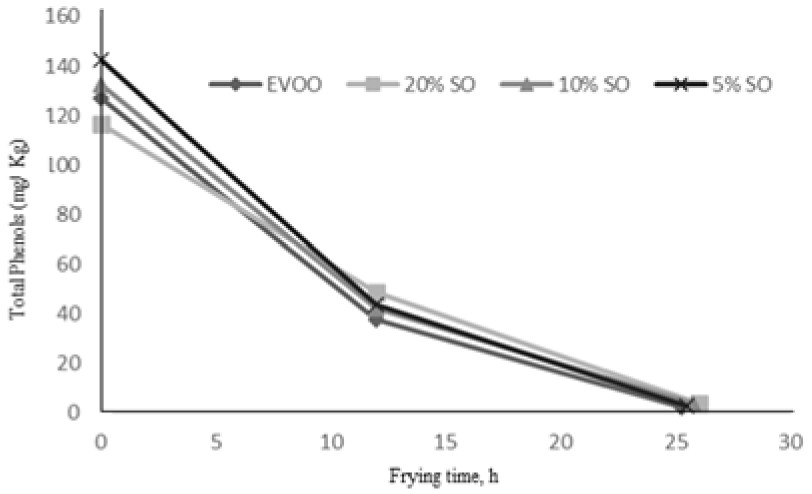

Figure S3: Total phenols (mg GA/Kg oil) during deep frying EVOO and blends with SO

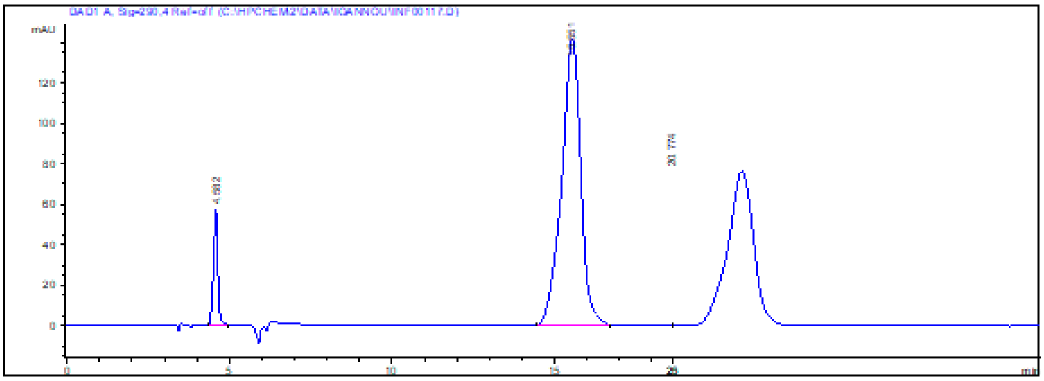

Figure S4: HPLC chromatogram of a mixed standard solution of sesamol, sesamin, and sesamolin.

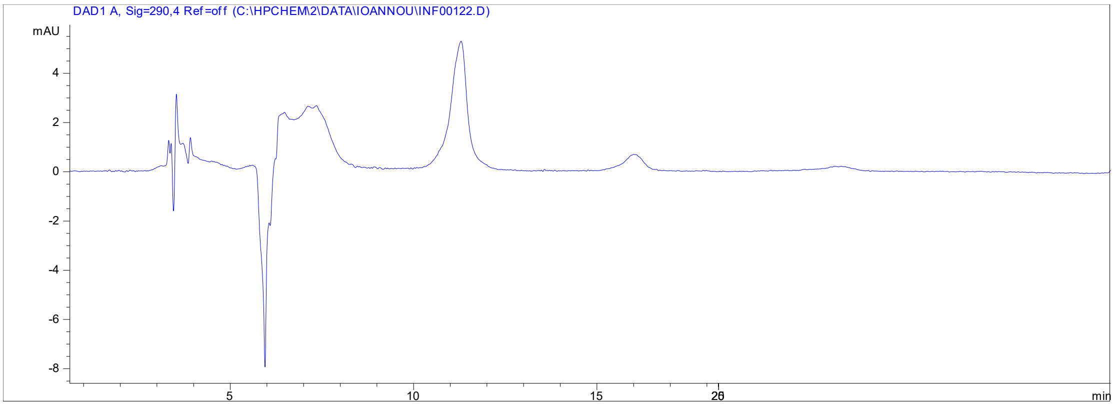

Figure S5: HPLC chromatogram of blend 20% v/v Sesame Oil in Olive Oil before frying.

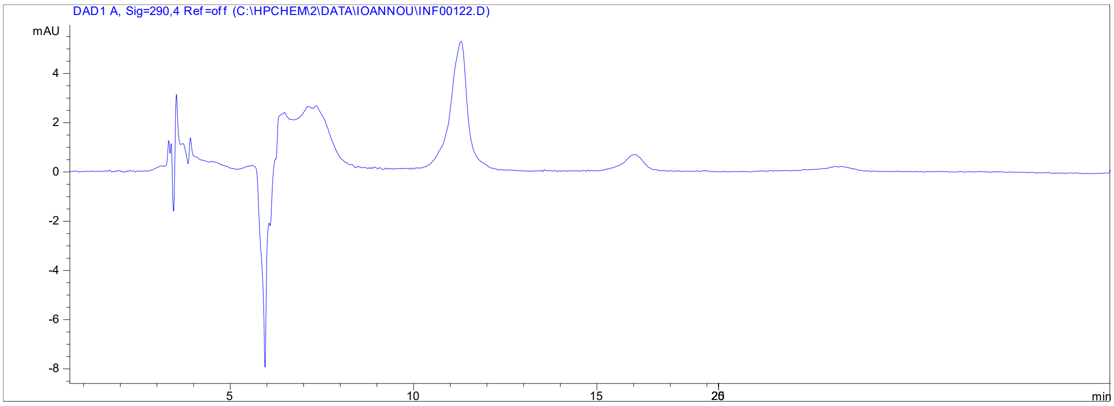

Figure S6: HPLC chromatogram of blend 20% v/v Sesame Oil in Olive Oil after 30 min frying.

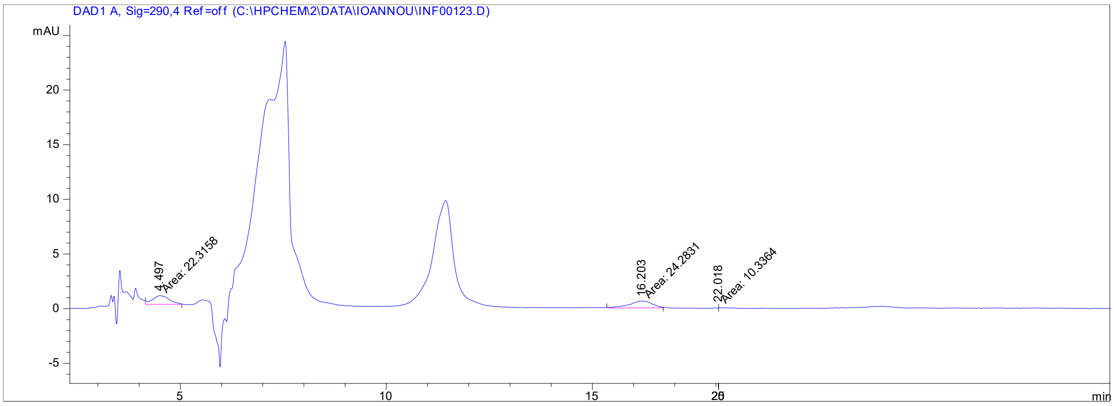

Figure S7: HPLC chromatogram of blend 20% v/v Sesame Oil in Olive Oil after 1h of frying.

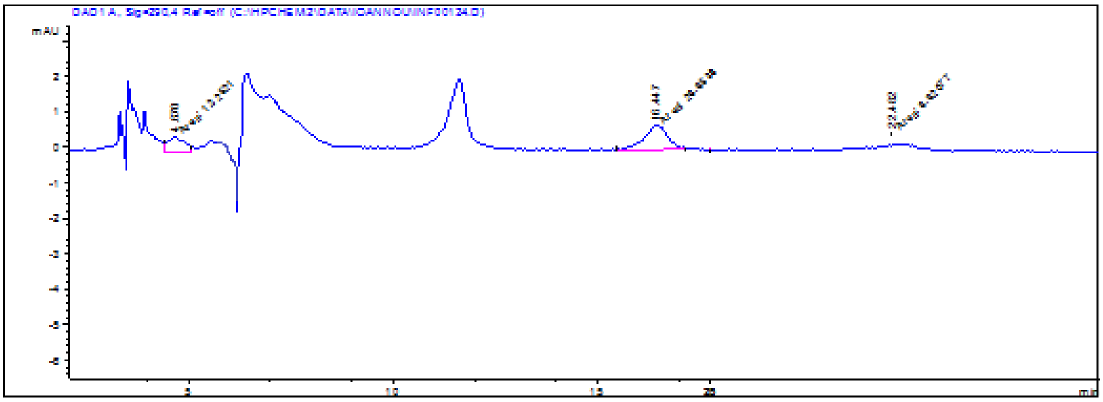

Figure S8: HPLC chromatogram of blend 20% v/v Sesame Oil in Olive Oil after 2h of frying.

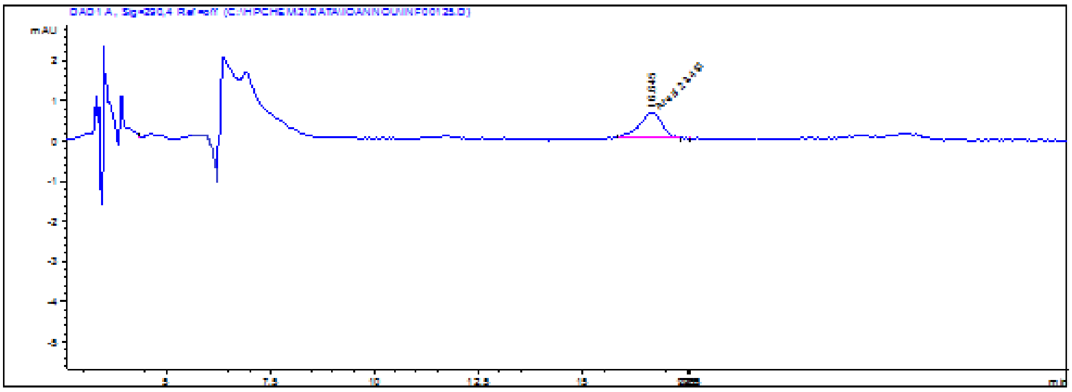

Figure S9: HPLC chromatogram of blend 20% v/v Sesame Oil in Olive Oil after 4h of frying.

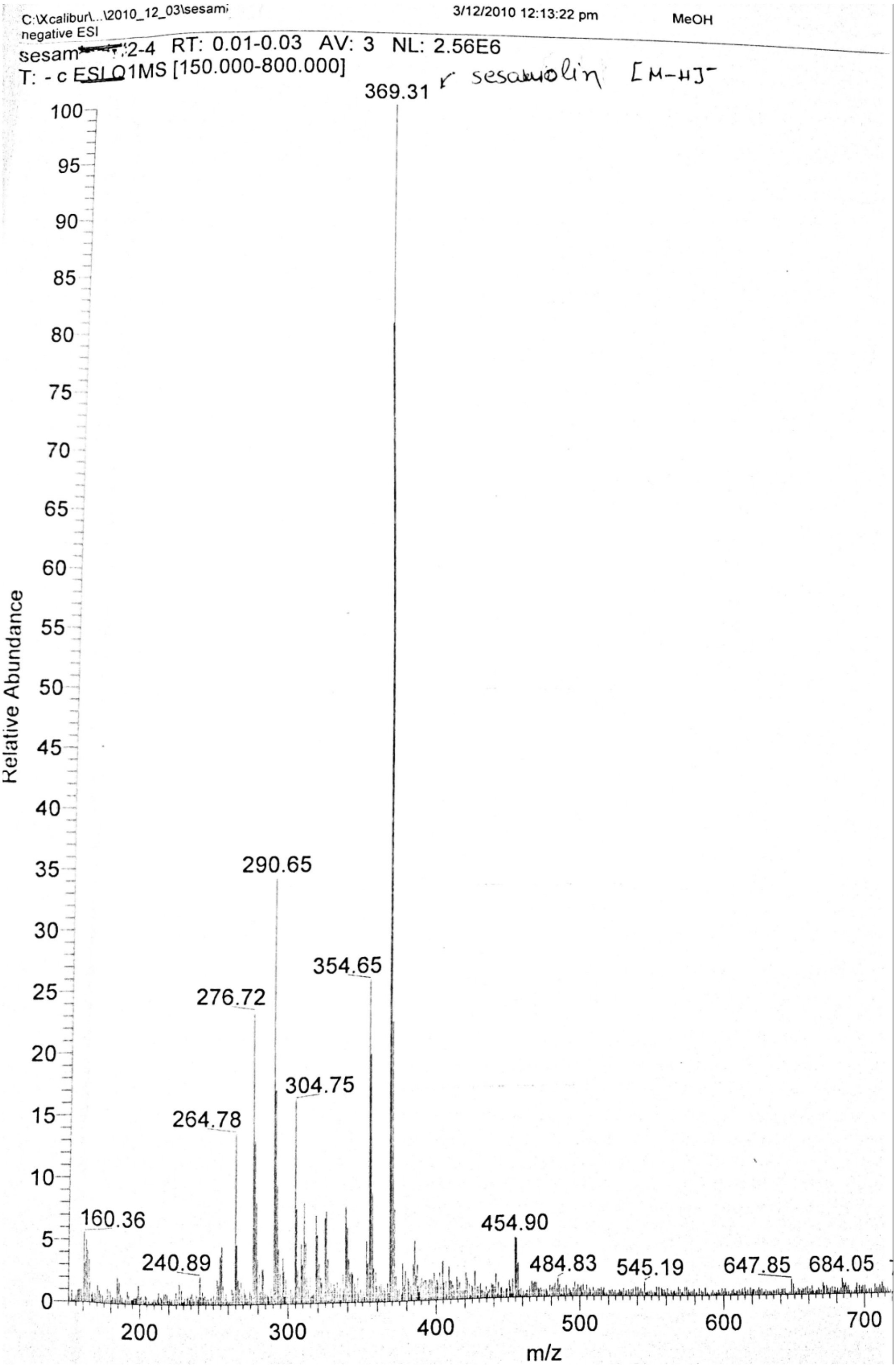

Figure S10: mass spectrum of Sesamolin

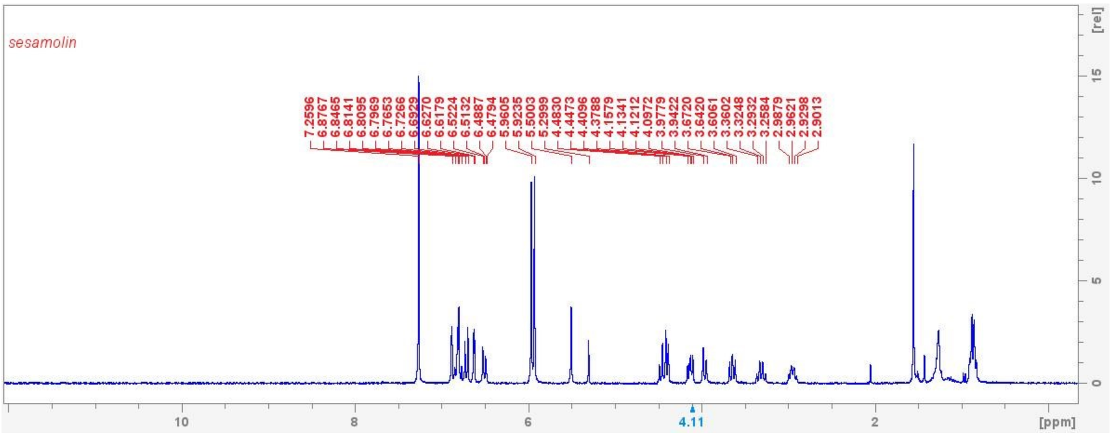

Figure S11 : ^{1}H- NMR spectrum of sesamolin.

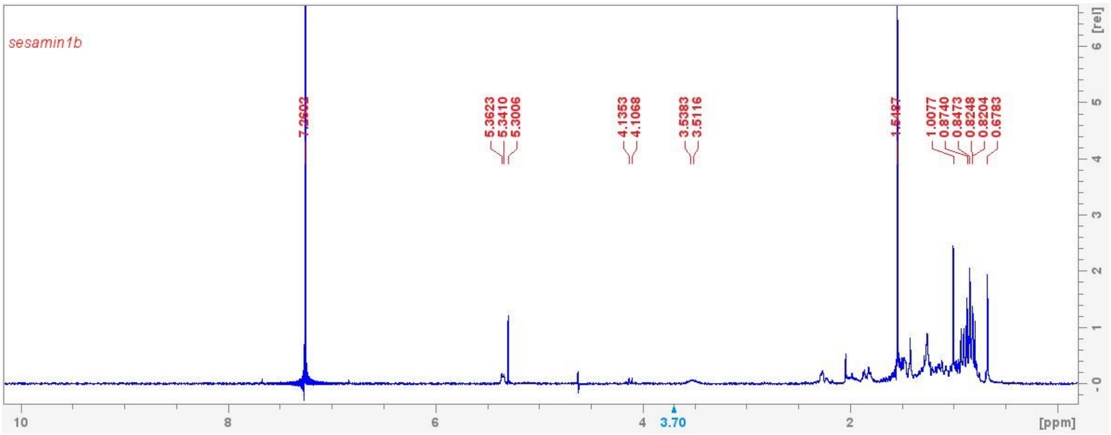

Figure S12 : ^{1}H- NMR spectrum of sesamin.
